# A draft genome sequence for the *Ixodes scapularis* cell line, ISE6

**DOI:** 10.12688/f1000research.13635.1

**Published:** 2018-03-08

**Authors:** Jason R. Miller, Sergey Koren, Kari A. Dilley, Derek M. Harkins, Timothy B. Stockwell, Reed S. Shabman, Granger G. Sutton

**Affiliations:** 1 J. Craig Venter Institute, Rockville, MD, 20850, USA; 2 Shepherd University, Shepherdstown, WV, 25443, USA; 3 Genome Informatics Section, Computational and Statistical Genomics Branch, National Human Genome Research Institute, Bethesda, MD, 20892, USA; 4 NBACC, Fort Detrick, MD, 21702, USA; 5 ATCC, Gaithersburg, MD, 20877, USA

**Keywords:** tick, genome, cell line, ISE6, Ixodes scapularis

## Abstract

**Background:** The tick cell line ISE6, derived from *Ixodes scapularis*, is commonly used for amplification and detection of arboviruses in environmental or clinical samples.

**Methods:** To assist with sequence-based assays, we sequenced the ISE6 genome with single-molecule, long-read technology.

**Results:** The draft assembly appears near complete based on gene content analysis, though it appears to lack some instances of repeats in this highly repetitive genome. The assembly appears to have separated the haplotypes at many loci. DNA short read pairs, used for validation only, mapped to the cell line assembly at a higher rate than they mapped to the *Ixodes scapularis* reference genome sequence.

**Conclusions:** The assembly could be useful for filtering host genome sequence from sequence data obtained from cells infected with pathogens.

## Introduction

The *Ixodes scapularis* embryonic 6 (ISE6) cell line is a widely used resource that is permissive to pathogens including human pathogens transmitted by ticks. Two decades ago, a collection of *I. scapularis* cell lines were derived from embryonated eggs including IDE lines derived from northern ticks and ISE lines derived from southern ticks ( Munderloh *et al.*, 1994). Recent proteomics analysis suggested that the ISE6 line is derived from neuronal cells ( Oliver *et al.*, 2015). ISE6 cells have been used to isolate and analyze bacterial pathogens including: the causative agent of human granulocytic ehrlichiosis (HGE) ( Munderloh *et al.*, 1999); *Borrelia burgdorferi*, the causative agent of Lyme disease ( Obonyo *et al.*, 1999); the causative agent of southern tick-associated rash illness (STARI) ( Varela *et al.*, 2004); and *Rickettsia felis*, the causative agent of spotted fever ( Pornwiroon *et al.*, 2006). ISE6 cells have been used to study viral pathogens including: Semliki Forest virus (SFV) and Hazara virus (arbovirus, family *Bunyaviridae*, genus *Nairovirus*) ( Garcia *et al.*, 2005); and Langat virus (LGTV), a Flavivirus ( Grabowski *et al.*, 2016). The cells have also been used to study RNAi and genome engineering in ticks; reviewed in ( Oliver *et al.*, 2015).

The *Ixodes scapularis* (black-legged tick) genome had been estimated to harbor 70% repeat content ( Ullmann *et al.*, 2005) when our lab participated in a community effort to sequence and assemble a tick genome. A reference sequence built from 3.8X Sanger sequencing and the Celera Assembler ( Istrail *et al.*, 2004; Myers *et al.*, 2000) software was fractured into 570,640 contigs (369,495 scaffolds) with a contig N50 of only 2,942 bp. The total contig span was 1.388 Gbp though the genome size was estimated at 2.1 Gbp. The assembly supported the annotation of 20,486 protein-coding genes and an extensive analysis of tick biology ( Gulia-Nuss *et al.*, 2016). The assembly is maintained at VectorBase ( Giraldo-Calderón *et al.*, 2015) under the name IscaW1.

A genome assembly for the ISE6 genome would assist investigations of ISE6 as a biological system. It would also provide a host subtraction tool for ISE6-based sequencing assays. Host subtraction is the bioinformatics process of filtering reads whose origin is host DNA and RNA ( Daly *et al.*, 2015). Host subtraction enriches the non-host component of sequence datasets and is especially attractive for assays involving high-throughput sequencing technologies that generate short reads in high volume where data reduction can realize cost savings. Following host subtraction, remaining reads can be mapped to references and counted, or used as queries to sequence databases, or assembled to reconstruct novel transcript or genome sequences. With an expectation that the IES6 genome would be as challenging as the tick genome, we sequenced IES6 with high coverage and long reads that, taken together, might generate a high quality reference genome assembly.

## Methods

### Cell growth

ISE6 cells were obtained from the American Type Culture Collection (ATCC), cell line CRL-11974, lot number 100005, patent 5,869,335. This cell line had been isolated from ticks collected in Georgia, USA. As generally described by ATCC technical bulletins and personal communications, cells were grown in Leibovitz L-15B media pH 7.0 ( Leibovitz, 1963) (ThermoFisher Scientific) supplemented with 80 mM glucose, 10% tryptose phosphate broth, 0.1% bovine lipoprotein cholesterol concentrate and 2% heat inactivated fetal bovine serum ( Munderloh & Kurtti, 1989). An addition of 0.7% non-essential amino acids (NEAA) concentrate (ThermoFisher Scientific) was also added prior to incubation. The cells were incubated in a gently shaking flask at 31°C with no C0 _2_.

### Sequencing

For short-read sequencing, genomic DNA was isolated from the cell line using a Qiagen genomic DNA isolation kit. Bioanalyzer analysis confirmed high molecular weight DNA was recovered. The library was size selected using Pippin Prep and prepared using the NextGen paired end barcoded genomic library construction protocol. Library quantification and normalization was performed by qPCR. The library was sequenced on the Illumina NextSeq 500 platform to generate 2x150 paired reads. Reads were demultiplexed which removed barcodes and sequencing adapters, and further treated with CutAdapt 1.8.1 to remove any remaining adapter.

For long-read sequencing, cells were grown until they attached to the flask. Genomic DNA was extracted from ISE6 cells using a Qiagen Genomic DNA isolation kit, stopping prior to the G2 isolation step. Frozen pellets and frozen cells were shipped to the Icahn School of Medicine at Mount Sinai for library construction and sequencing using standard SMRTbell template preparation kits (Pacific Biosciences). A total of 52 SMRTcells were run on the PacBio Sequel platform using standard PacBio protocols.

### Assembly and analysis

The long reads were corrected and assembled with the Canu assembler ( Koren *et al.*, 2017) version 1.6. Canu was run with the SGE grid engine and Java 1.8 using default parameters except: minOverlapLength = 1000 bp, corMhapSensitivity = "low", and genomeSize = 1 Gbp. The contig consensus sequences were polished using SMRT Link version 5.0.1.9585 which includes Arrow version 2.2.1, blasr 5.3, and pbalign 0.3.1 (Pacific Biosciences).

Short reads were mapped with bowtie2 ( Langmead & Salzberg, 2012) version 2.2.5 using either end-to-end or local-alignment mode as indicated in the text. Using default settings, the mapper reported at most one mapping per read and reported read maps individually though it used reads as pairs to select alignments. Mappings were analyzed with samtools ( Li *et al.*, 2009) version 1.2.1 and bedtools ( Quinlan, 2014; Quinlan & Hall, 2010) version 2.26. K-mers were counted using Jellyfish ( Marçais & Kingsford, 2011) version 2.2.6 for several values of K: 11, 15, 21, 41, 51, and 61. Each computed K-mer histogram was analyzed with GenomeScope ( Vurture *et al.*, 2017). Single-copy gene analysis used BUSCO ( Simão *et al.*, 2015) version 3.0.2 with Arthropoda OrthoDB version 9. Alignments between the tick and cell line assemblies were computed on contigs from each assembly using nucmer, the local aligner in the MUMmer package ( Kurtz *et al.*, 2004) version 3.1. The BLAST analysis used TBLASTN in NCBI BLAST+ ( Camacho *et al.*, 2009) version 2.2.31. The Ixodes protein predictions were downloaded from UniProt in Nov 2017.

## Results

### Assembly

PacBio sequencing yielded 192.5 Gbp in 27.3 million unpaired reads, providing approximately 92X coverage of the estimated 2.1 Gbp tick genome. This dataset included 190.7 Gbp in reads >= 1 Kbp and 115.9 Gbp in reads >= 10 Kbp. To overcome high base call error observed in single-molecule long-read data, the long reads were subjected to the Canu correction process which filtered, trimmed, and polished reads based on alignment with other long reads ( Koren *et al.*, 2017). Correction yielded 36.7 Gbp in 2.1 million reads with a 17,680 bp N50. Read counts are shown in Table S1. Read length distributions, before and after correction, are shown in Figure S1.

The corrected long reads were assembled in isolation with the Canu long-read assembler ( Koren *et al.*, 2017). The initial assembly, named Ise6_asm0, contained 18,717 contigs. The uncorrected long reads were used to polish the contig consensus sequences using the Arrow process. This was run in two iterations to produce assemblies Ise6_asm1 and Ise6_asm2 respectively. The released assembly, named Ise6_asm2, contained 2,691,078,110 bases in 18,717 contigs with a 269,660 bp contig N50. Statistics for each assembly are shown in Table S2.

### Assembly quality assessment

Since the tick contigs are generally smaller than the cell line contigs, it seemed likely that some tick contigs would be wholly contained by cell line contigs. The IscaW1 and Ise6_asm0 assemblies were compared with the MUMmer local alignment software; see Table S3. A high-confidence alignment subset (filtered with delta-filter -1) was used. The average identity of aligned bases was 96.58%. Both assemblies were almost fully covered by alignments. Using alignments of 1 Kbp or more, IscaW1 contigs with at least one alignment made up 78.6% of IscaW1 bases. The set of IscaW1 contigs with at least 90% coverage in any one alignment made up 35.5% of IscaW1 bases.

To provide an orthogonal dataset for assembly assessment, the genome was also sequenced on a short-read platform. Illumina sequencing yielded 25 Gbp in 171 million 2x150 bp paired reads, providing approximately 24X short-read coverage of the 2.1 Gbp estimated genome size of the tick. Comparative map statistics are shown in Table S4. When paired reads were mapped to Ise6_asm0 using a local alignment algorithm, 97.83% of reads mapped as a concordant pair. For comparison, when the reads were mapped to the IscaW1 tick reference assembly, 91.68% of reads mapped as a concordant pair. This result indicates that the ISE6 assembly is more representative of ISE6 genome structure than the *Ixodes* reference.

For consensus quality assessment, the paired reads were mapped to Ise6_asm2 and IscaW1 contigs using a more stringent, global (end-to-end) alignment algorithm; see Table S4. Among these alignments, the read sequence disagreement with the contig consensus was 1.79% for Ise6_asm2 and 5.03% for IscaW1. This demonstrates that the ISE6 consensus is more representative of ISE6 genome sequence than the *Ixodes* reference.

The rates of concordant pair mapping to zero, one, or multiple sites were 23%, 29%, and 48% respectively for Ise6_asm2 and 44%, 30%, and 25% for IscaW1; see Table S4. Thus, by paired-read mappability, both assemblies contain 29%–30% unique sequence while the ISE6 assembly captures an additional 23% of reads and these align to repeat sequences in the assembly.

The global alignment 23% unmapped rate in Ise6_asm2 is an order of magnitude larger than the unmapped rate among the local alignments. It is possible that the long and short read sequencing captured genuine differences at unstable regions of the cell line genome. It seems more likely that the genome harbors repeat instances that are similar-but-not-identical to those in the assembly.

Using the global alignments and accepting all mapped reads (whether mapped as a pair or not), the Ise6_asm2 assembly mapped 81% of reads while IscaW1 mapped 65%. Thus, the Ise6_asm2 assembly outperformed the IscaW1 assembly as a host subtraction tool using pairwise local, pairwise global, and read-wise global alignments.

The assembly was assessed for completeness using gene content analysis. The latest UniProt protein predictions on the IscaW1 tick genome assembly were used as TBLASTN query sequences against the cell line assembly. Out of 20,473 predicted proteins: 20,290 (99.1%) had at least one hit in Ise6_asm0 while 183 predictions had no hit. The Ise6_asm2 assembly was analyzed for gene content using the BUSCO collection of genes thought to be single-copy in arthropod genomes; Table S5. Of 1066 genes searched, 1.4% were fragmented, 3.6% were missing, and 95% were complete. These results indicate that the assembly is fairly complete for single-copy genes.

### Genome size analysis

The Ise6_asm2 contig span is 2.8 Gbp which exceeds the 1.4 Gbp contig span of the IscaW1 tick reference assembly as well as the 2.1 Gbp estimated genome size for tick. The discrepancy could be due to several factors. It is possible that the cell line genome is larger than the tick genome, or that the assembly contains dual representations of heterozygous loci that assembled separately, or that the IscaW1 reference assembly underrepresents repeats present in the tick and ISE6 genomes. These possibilities were explored with several analyses.

K-mer analysis ( Vurture *et al.*, 2017) provides an assembly-free genome size estimate extrapolated from the frequency distributions of short, contiguous sequences extracted from the sequencing reads and counted using an exact-match algorithm ( Marçais & Kingsford, 2011). In K-mer analysis of our short-read data, the observed distribution could not be fit to a model. Analysis of the corrected long read K-mers was similarly inconclusive. As illustrated by the representative plot in Figure S2, the distribution does not include a strong peak other than the mode at 1X. These results are possibly due to the low coverage in both datasets, which would be especially low if heterozygous haplotypes were represented separately in contigs.

Next, genome size estimation was attempted using coverage analysis of paired reads mapped to contigs. Pairs mapped end-to-end provided 39.9 Gbp of mapped bases. In the distribution of read coverage per contig base, there is a smooth peak with a 9X mode and a tail at higher coverage; see Figure S3. Using the mapped base count divided by 9X to represent the average coverage of unique sequence in the genome, the extrapolated genome size is 4.43 Gbp, which is about twice as large as the tick estimated genome size. This suggests that the assembly process separated the haplotypes of a 2.22 Gbp diploid genome.

**Figure 1.**
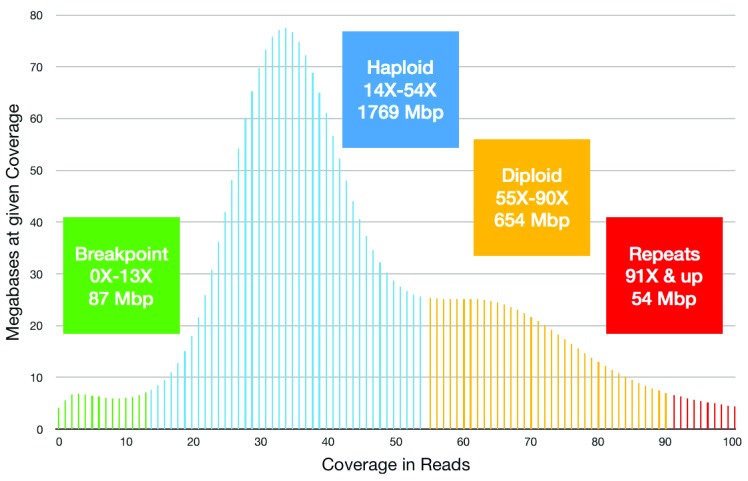
Long read coverage of the cell line assembly. Read coverage per base was computed by mapping uncorrected long reads to assembled contigs. Colors were added to highlight the following interpretation. Green: the minor peak of low-coverage bases suggests 87 Mbp of contig bases lies near breakpoints such as contig ends and false joins. Blue: the dominant peak, with mode at 34X, suggests that 1769 Mbp of the assembly is haplotype-separated sequence possibly representing 885 Mbp of the diploid genome. Yellow: the shoulder near 64X suggests that about 654 Mbp of the diploid genome is captured as diploid-consensus sequence. Red: high coverage (including bases not shown with coverage over 100X) suggests that under-represented genomic repeats occupy 54 Mbp of the assembly but more of the genome.

Long-read coverage was analyzed next. The uncorrected long reads had been mapped to Ise6_asm1 contigs for the final iteration of Arrow consensus polish. Based on those results, the per-base coverage peaks at 34X with a shoulder at almost twice that level; see Figure 1. This suggests that 34X represents the coverage mode for haplotype-separated sequence. With 175.48 Gbp in mapped reads, 158.47 Gbp of read sequence aligned to 151.93 Gbp of contig sequence. These mapping and coverage results combine to indicate 151.93 / 34 = 4.47 Gbp size for the combined haplotypes (diploid) and 2.24 Gbp for the haploid genome. Thus, the completeness of the 2.8 Gbp assembly is uncertain and the assembly appears to harbor double-representation of loci that are haplotype-separated and under-representation of genomic repeats, as indicated in Figure 1.

Local alignments between assemblies were examined for support of the haplotype separation hypothesis. The cell line Ise6_asm0 contigs were aligned to the tick reference IscaW1 contigs using the nucmer local alignment software. As shown in Figure S4, the largest IscaW1 contig aligns full-length to two contigs of Ise6_asm0. The full set of alignments was filtered to retain one best alignment per Ise6_asm0 position (with delta-filter -q) and to retain only IscaW1 contigs with at least 50% coverage in one such alignment. Using these alignments to distinguish IscaW1:ISE6 multiplicities, 43% of IscaW1 bases are in contigs with 1:1 relation to ISE6, while 17% of bases are in contigs with 1:2 relations, and 3% of bases are in contigs with relations of 1:3 or higher; see Table S3. Not all contigs could be categorized. Further work would be required to fully partition and phase all duplicated contigs with high confidence.

Of 1066 BUSCO genes searched against Ise6_asm2, 67% were complete and single-copy, and 28% were complete and duplicated. This indicates that almost a third of single-copy genes are duplicated in the assembly, presumably due to haplotype separation. If this rate of sequence duplication applies to the assembly overall, the 2.67 Gbp assembly indicates a 2.29 Gbp genome size.

### Non-Ixodes sequence

During the assembly submission to GenBank, NCBI flagged one contig for sequence similarity to *Rickettsia*, a genus of endosymbionts common to *Ixodes scapularis* ( Gillespie *et al.*, 2012; Labruna *et al.*, 2007; Steiner *et al.*, 2008; Zeringóta *et al.*, 2017). The long read coverage in the flagged region differs from the flanking regions; see Figure S5. The contig was split and re-submitted without the flagged region.

## Discussion

The ISE6 cell line, derived from the Lyme disease tick *Ixodes scapularis*, is widely used but has so far lacked a genome reference sequence. With this report, the genome has been sequenced, assembled, and released. The genome sequence was generated from high coverage in PacBio Sequel long reads, assembled with the Canu assembler, and polished with Arrow. Gene content analysis indicated that the assembly is largely complete though read mapping analysis indicated that some genomic repeats are under-represented in the assembly. Read mapping and single-gene analysis indicated that portions of the genome are represented as a diploid consensus while other portions are represented in haplotype-separated copies. The new assembly provides a more accurate representation of the cell line genome compared to the previous closest reference, which was an assembly of the *I. scapularis* tick genome. In our mapping of cell line gDNA short read pairs that were not used for the assembly, the cell line assembly was more effective for identifying host reads compared to the tick reference. Thus, the new assembly provides a resource for analysis of the cell line and for host subtraction to assist the detection of pathogens present in the cells.

Comparable genome size estimates were obtained by three methods. Short-read coverage analysis indicated 2.22 Gbp. Long-read coverage indicated 2.24 Gbp. Single-copy gene analysis indicated 2.29 Gbp. The tick genome was previously estimated to be 2.1 Gbp so the cell line may harbor some ISE6-specific sequence. Identification of such sequences is left for future work. Our local alignments of the tick and ISE6 assemblies covered nearly all of both assemblies, so any cell-line-specific sequence is likely to involve genomic repeats.

We hope to enhance the assembly resource in several ways. We hope to generate a second version of the sequence using different assembly parameters. A larger genome size estimate, in particular, should lead to higher generated coverage in corrected reads. Additional corrected reads would necessarily be shorter than the ones used here, but they could provide additional depth for repeat detection and repeat resolution. We hope to provide gene annotation, repeat content analysis, and more specific haplotype separation analysis of the second assembly.

## Data availability

The gDNA Illumina and PacBio reads are available at NCBI SRA under BioSample SAMN06329993. This entire assembly has been deposited at GenBank under the accession GCA_002892825.1. Whole Genome Shotgun sequencing project is available under accession PKSA00000000.

## Supplementary material

**Figure S1. Length distributions for PacBio long reads before (blue) and after (green) correction by the Canu assembler.** The comparison indicates that the correction step removed most short reads while reducing the number and lengths of long reads. Each blue or green vertical bar represents the number of reads in a 10 bp window. Reads longer than 30 Kbp (not shown) provided 1,623,767,067 bp in 47,967 reads before correction and 333,923,002 bp in 10,211 reads after correction.

**Figure S2. K-mer frequency distribution plotted by GenomeScope from K-mers found in PacBio long reads by Jellyfish with K=41.** Similar plots were generated by GenomeScope for selected values of K ranging from 11 to 61 from the corrected long reads and also the (uncorrected) short reads. The distribution is dominated by K-mers that occur once (coverage = 1X), which can be attributed to random base call error.

**Figure S3. Histogram of short-read coverage of large contigs.** The mode of the distribution is at 9X. The minor peak at 0X indicates uncovered contig bases, which are frequent at contig ends. The pseudo-peak at 51X represents all bases covered at 51X or greater. Reads were mapped to all contigs but only large contigs (length >= 10Kbp) were plotted here. The mapping used end-to-end alignments and reported at most one map per read. The mapping used reads as pairs but reported all mapped reads. A map report produced with ‘samtools stats’ reported 277,543,724 reads mapped including 261,690,992 properly paired in the mapping, and 39,913,302,735 based mapped and no clipping. Assuming 9X is the average read coverage of unique sequence in the genome, the genome size is estimated as 278 Gbp / 9X = 4.4 Gbp, or roughly twice the tick estimated genome size. This suggests that the assembly captures many diploid loci twice and that 9X is the average coverage of a haplotype-separated sequence in the assembly.

**Figure S4. Alignment of two ISE6 contigs to one IscaW1 contig.** Contig ABJB010274751.1, the longest IscaW1 contig, has length 117,687 bp and is represented full-length on the X-axis. This contig has nearly-complete, full-length coverage in alignments to two ISE6_ASM1 contigs, each represented full-length on the Y-axis. Alignments were computed with nucmer and plotted with mummerplot using minimum length 1000 bp. ISE6_ASM1 contig tig00013410 (top) has length 309,049 bp and 19 local alignments including the 13 large alignments shown. ISE6_ASM1 contig contig tig00009157 has length 719,239 bp (bottom) and 30 local alignments including the 20 large alignments shown.

**Figure S5. Sequence identified as *Rickettsia* has unusual read coverage distribution.** As part of the Ise6_asm2 assembly submission to GenBank, NCBI flagged one contig for having sequence similarity to *Rickettsia*, A search using NCBI megablast and the nt database confirmed that tig00009859, of length 618,170 bp, had a portion of sequence that was most similar to *Rickettsia* (various species). Uncorrected PacBio reads had been mapped to the Ise6_asm1 version of the assembly as part of the Arrow polish process that generated Ise6_asm2. Read coverage at every base was extracted from that mapping using samtools. Read coverage was plotted for the three coordinate ranges 1–250,000 (orange, upstream), 250,000–350,000 (blue, contig middle), and 350,000–600,000 (downstream, green). The flagged region falls within the contig middle coordinates. Because the contig middle appeared to have a different coverage profile from both flanking sequencings, the flagged region (268 Kbp to 330 Kbp) of the contig was removed and the upstream and downstream contigs were re-submitted to GenBank.

**Table S1. ISE6 sequencing results.**

**Table S2. ISE6 contig size statistics for several assemblies.**

**Table S3. Nucleotide local alignments between the contigs of IscaW1 and Ise6_asm0.** Alignments were generated and analyzed with the MUMmer package including the delta-filter alignment filter.

**Table S4. Alignment rates for paired reads mapped to contigs.** Short reads were mapped with bowtie2 in local mode or global (end-to-end) mode.

**Table S5. Single-gene content analysis with BUSCO.** Analysis of Ise6_asm2 using the Arthropoda OrthoDB version 9 single-copy genes.
