# Supplementary figures and images for: A draft genome sequence for the Ixodes scapularis cell line, ISE6
Source: F1000Res. 2018 Mar 8;7:297. [Version 1] doi: 10.12688/f1000research.13635.1 (PMC5883391; doi:10.12688/f1000research.13635.1)

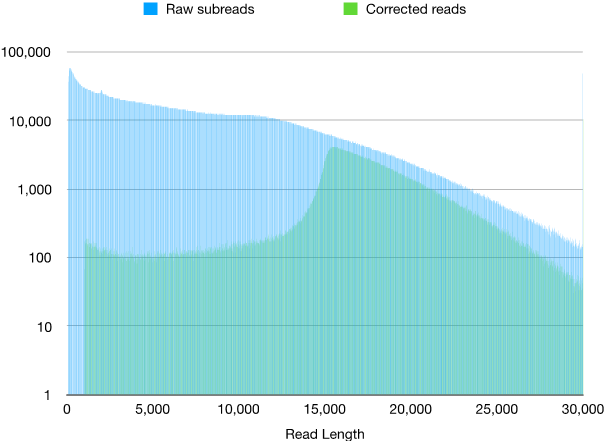

Supplement: Supplementary file 1 [file f1000research-7-14813-s0000.tgz › 15dabbe9-a98b-4861-84a6-c20171e4adf5.tif]

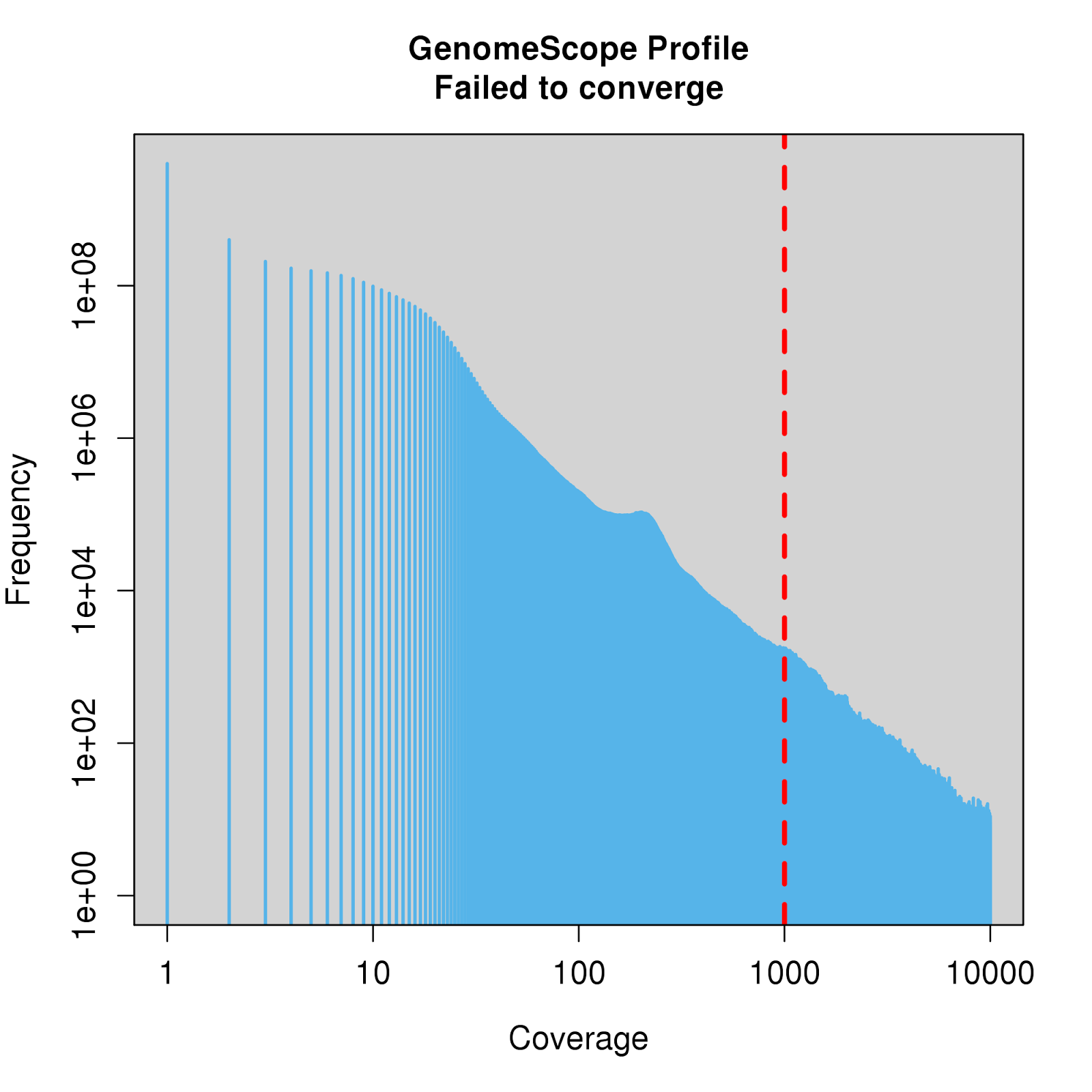

Supplement: Supplementary file 2 [file f1000research-7-14813-s0001.tgz › 2018a51b-3039-4863-8a99-ec1299310853.tif]

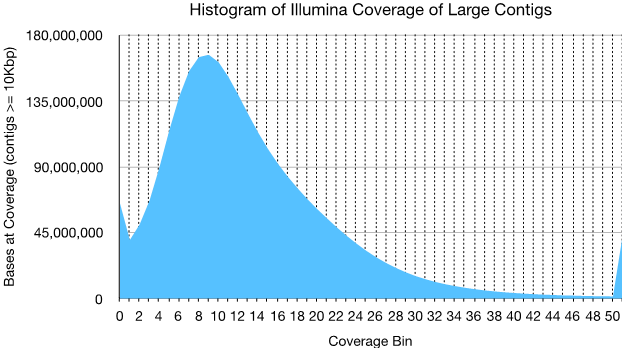

Supplement: Supplementary file 3 [file f1000research-7-14813-s0002.tgz › 7eda98cd-dc62-4ac4-bc4f-897ddfdbe514.tif]

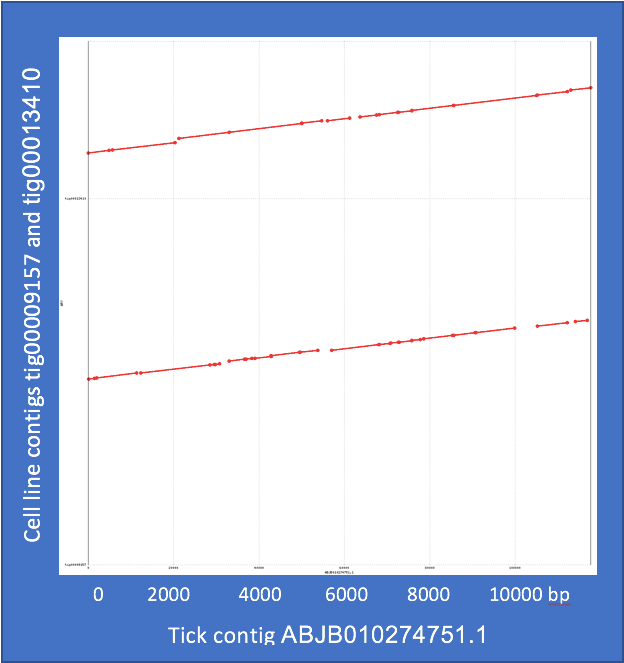

Supplement: Supplementary file 4 [file f1000research-7-14813-s0003.tgz › df9f42b7-444a-4e1d-a8bb-aa9fae17a9de.tif]

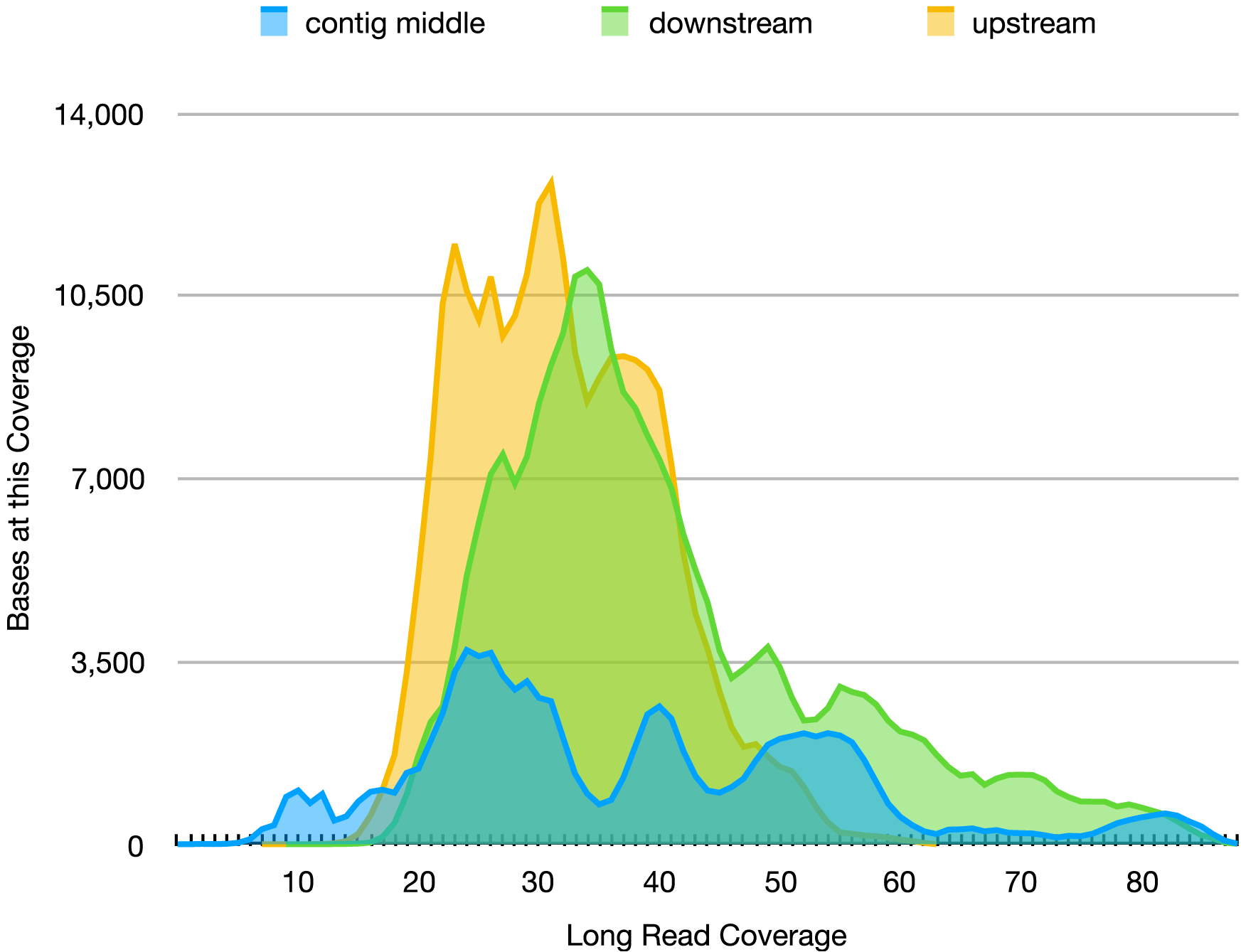

Supplement: Supplementary file 5 [file f1000research-7-14813-s0004.tgz › 505ea76b-2dbe-44f6-a6bd-da61e3eaaa02.tif]
