# Supplementary material for: A draft genome sequence for the Ixodes scapularis cell line, ISE6
Source: F1000Res. 2018 Mar 8;7:297. [Version 1] doi: 10.12688/f1000research.13635.1 (PMC5883391; doi:10.12688/f1000research.13635.1)
Supplement: Peer review history

# Peer review: Referee response for version 1

**DOI:** 10.5256/f1000research.14813.r31976

**Published:** 2018-04-03

**Authors:** Chan Heu

**Affiliations:** 1 Pennsylvania State University, State College, PA, USA

The authors assembled a draft genome of ISE6 using long-read sequencing. In addition, they used short-read sequencing to validate and distinguish the ISE6 assembly through differences in genome size and mapping coverage from the already sequenced, albeit incomplete, *Ixodes scapularis* genome. The primary aim is to generate the genome of ISE6 for host genomic for potential application such as the subtraction from genomic analysis of pathogens and/or symbionts cultured in ISE6. The authors demonstrated very well that the draft genome of ISE6 showed more coverage than the tick genome. It was also a great approach to use three different methods to estimate the genome size.

 There were a few areas that could have been improved upon. This article would have benefited from demonstrating if there is any difference in using the ISE6 genome assembly vs. the tick genome for subtracting host genomic background, or in some other application in which using the assembled ISE6 genome would be more beneficial than the tick genome. In addition, the authors could discuss further the origin of the contig that was similar to *Rickettsia*. For instance, the authors could hypothesize whether this contig came from a lateral gene transfer event, rickettsial contamination of the starting material, or technical error in the assembly process. Nevertheless, having a draft genome of ISE6 will be useful for in vitro research involving tick-associated bacteria, protozoans, and viruses.

I have read this submission. I believe that I have an appropriate level of expertise to confirm that it is of an acceptable scientific standard.

# Peer review: Referee response for version 1

**DOI:** 10.5256/f1000research.14813.r31696

**Published:** 2018-03-22

**Authors:** Scott Emrich

**Affiliations:** 1 University of Tennessee, Knoxville, TN, USA

Miller et al. present a long-read based assembly of the ISE6 tick cell line. Although mostly in the background/discussion sections of this paper, the Ixodes genome has been hard to assemble given a large number of repeats and heterogeneity between individuals. Similar to other recent long read efforts of mosquitoes, continuity / completeness has been improved by at the cost of residual haplotypes; only ~66% of the genome is single copy. 

 On the one hand this does not impact the main use case of this effort, namely an improved reference for host extraction. As a nice twist the authors included an Illumina experiment, for example, and showed more host (tick) DNA aligned to their reference. Given this cell line is most used for pathogen-related work this is a valuable contribution even though the assembly itself contains haplotypes.

 On the other hand, this paper in of itself does not really provide any new solutions or even discussion on how to improve assembly or a reference tick genome, which makes it more of a data note (and fine for F1000Research). For example, getting DNA from a single individual is sometimes hard so other projects have resorted to using full sibs. Given that the cell line was derived from ticks (plural) is it even feasible to dilute the cell line to get what amounts to clonal flasks and try and sequence that? That would not help the repeat issue but may reduce the amount of heterogeneity.

 I have a few remaining specific questions/comments listed below:

 1.  On page 3 you mention because 6.7% more pairs are concurrent you state "This result indicates that the ISE6 assembly is more representative of ISE6 genome structure than the Ixodes reference." This is not wrong, but I have two followup questions.  First, in the next section you observe that there is over 5% divergence between your line and the Ixodes reference (which makes sense). Could this affect your short-read alignment parameters and cause less pairs to map? Second, what if your contigs are 2X bigger that map these additional pairs but they are repeated in your assembly but not the genome (residual heterogyosity). I guess my issue is using "structure" vs. a more generic "contents" given the issues reported in the build.

 2.  Similarly, you state on page 3 that the 25% of reads that map to multiple sites are repeats, but couldn't they map to un merged alleles given the later results in the manuscript? I realize repeats is ambiguous and the haplotype issue is discussed later but maybe generalize to "repeated sequences present in the assembly" (or similar) to allow for assembly artifacts and "real" repetitive sequences present in tick

 3.  I was curious as to the BUSCO results of the old assembly ... the Ixodes reference was done long enough ago I am not sure they would have used it and a reader shouldn't have to look it up. In short, if we ignore the allele duplication issue are genes assembled better? The answer was yes for Aedes and I assume so here but it is unclear.

I have read this submission. I believe that I have an appropriate level of expertise to confirm that it is of an acceptable scientific standard.
